# Effects of Harvesting Time on Fruit Development Process and Oil Content of Selected Iranian and Foreign Olive Cultivars under Subtropical Conditions

**DOI:** 10.3390/plants12142737

**Published:** 2023-07-23

**Authors:** Narjes Fahadi Hoveizeh, Rahmatollah Gholami, Seyed Morteza Zahedi, Hojattollah Gholami, Petronia Carillo

**Affiliations:** 1 Department of Horticultural Science, College of Agriculture, Shahid Chamran University of Ahvaz, Ahvaz 61357-83151, Iran; narjes.fh64@yahoo.com; 2 Crop and Horticultural Science Research Department, Kermanshah Agricultural and Natural Resources Research and Education Center, AREEO, Kermanshah 67145-1661, Iran; 3 Department of Horticultural Science, Faculty of Agriculture, University of Maragheh, Maragheh 83111-55181, Iran; s.m.zahedi@maragheh.ac.ir; 4 Department of Plant Protection, Faculty of Agriculture, University of Kurdistan, Kurdistan 66177-15175, Iran; h.gholamiii2@yahoo.com; 5 Department of Environmental, Biological and Pharmaceutical Sciences and Technologies, University of Campania Luigi Vanvitelli, 81100 Caserta, Italy

**Keywords:** high temperature, climate change, oil accumulation, dry matter, pomological attributes

## Abstract

Climate change and rising global average temperatures across the year may strongly affect olive fruits’ development process and their oil yield and quality. There is therefore an urgency to take immediate actions to characterize the wide variability of cultivars in order to identify those with a stable response to high temperatures, particularly in areas like the west of Iran, which is characterized by a warm summer continental climate. The objective of this study is to investigate the process of fruit development and oil accumulation in response to high summer temperature conditions in a set of four Iranian olive cultivars (Shengeh, Roughani, Zard Aliabad, and Dezful) in comparison with four foreign olive cultivars (Konservolia, Sevillana, Manzanilla, and Mission) in seven various harvesting times (20 July, 5 and 20 August, 5 and 20 September, 6 and 21 October). The obtained results evidence a significant positive correlation between fruit dry matter and oil content. High temperatures reduced the oil and dry matter accumulation in the second half of the summer, with severe thermal conditions adversely affecting oil synthesis. Paramount variations were observed among the cultivars regarding oil accumulation, dry matter, and pomological attributes. All of them showed the highest oil content at the last harvest. Among all analyzed varieties, Roughani showed the highest tolerance and adaptive capacity to high temperatures as it accumulated the greatest amount of dry matter as well as oil content in all of the harvesting times, demonstrating a positive correlation between these two traits. Although Shengeh showed the lowest oil content on a dry and fresh weight basis at the first harvesting time, this cultivar generally presented higher fruit development attributes than the other cultivars, highlighting that it benefits from a high temperature.

## 1. Introduction

Olive (*Olea europaea* L.) is a perennial evergreen tree with a wide geographical distribution notwithstanding its Mediterranean origin. In fact, due to its valuable oil, olive culture in Iran has expanded during the past two decades [1]. Approximately 90% of the world’s olive production is devoted to oil extraction, and the remaining 10% is devoted to table olives [2]. During the past few decades, the growing considerable awareness about the nutritional value of olive oil contributed to the expansion in the olive tree cultivation regions over the world [3,4]. Olive oil has proven to have health advantages due to its high value of monounsaturated oleic fatty acids, which represent 55–83% of the total olive oil. More precisely, the low content of saturated fatty acids in olive oil and the high level of monounsaturated fatty acids, in addition to the antioxidant and anti-inflammatory properties, make olive oil a healthy source of fat, which can decrease cardiovascular disease risks [3].

As in other drupes, the olive fruit size and weight curve are characterized by an expected double sigmoid pattern. After anthesis, mesocarp growth occurs only due to cell expansion, and for 6 weeks after full bloom, cell division ceases. After fruit set, the fruit weight elevates linearly; however, during pit hardening, it stays steady and subsequently increases again before ripening [5]. Oil synthesis in olive fruits substantially occurs after pit hardening in the parenchymatic cells of the mesocarp, and oil accumulation becomes the major sink of assimilates in the fruits [6].

The common areas for producing olive oil in Iran are characterized by mild winters and dry and long summers, during which fruit growth and oil accumulation in olive occur [7]. However, the increase in average temperatures during the summer season may adversely affect olive floral differentiation and fertilization, causing pistil abortion and, consequently, a decline in olive fruit set [8]. Indeed, the rate of oil accumulation in olive fruit over time is largely affected by external conditions as well as internal factors. In particular, the oil content is sensitive to temperature fluctuations, and by increasing the mean temperature between 16 °C and 32 °C, the oil content declines [9]. Temperatures above 25 °C caused a significant reduction in the olive fresh weight of the fruit as well as the fruit oil content [10]. As mentioned above, oil accumulation, as the most critical stage of olive fruit development, happens between pit hardening and fruit ripening. In the northern hemisphere, this period of time falls in the second half of the summer. Oil accumulation lasts approximately 8 weeks during the summer and fall, and after that, slows down over fruit ripening [5]. To obtain the best quality olive oils, the most favorable harvesting time must be defined for each site separately in dependence on different thermal regimes. The best quality olive oil is obtained in the sites with higher rainfall and lower temperatures, when the fruits are yellow-green and ripe [11]. Higher temperatures contribute to promoting vegetative growth but have a negative effect on the oil content; therefore, during an extremely dry and warm summer, oil accumulation occurs very slowly [12]. Moreover, oil accumulation, as a complicated process, takes place depending not only on the environment, but also on the cultivar [13]. A high temperature has a permanent negative impact on the olive oil quantity and quality, especially if this external condition occurs early. This damaging effect depends on the growth stage at which the plants are exposed to a high temperature, the severity of the stress, and the cultivar [14]. The influence of a high temperature on the oil content seems to be dramatically genotype dependent. Different olive varieties react to an extreme of the environment in a genotypic particular manner. Substantially, the early difference in fruit size among various cultivars is a result of the different rates of cell division according to genetic capacities. Obviously, the characterization of growth development and oil accumulation capacity over a wide range of cultivars and the identification of heat-resistant genotypes is the first step to olive breeding, especially in extreme heat conditions. Additionally, the elucidation of special response mechanisms adopted by tolerant cultivars opens the way toward the breeding of new olive cultivars that provide the possibility of olive cultivation in critically hot regions [15].

The assessment and evaluation of oil accumulation and growth parameter (fruit length, diameter and volume, pit length and diameter, fruit fresh weight, fresh and dry pulp weight, fresh and dry pit weight, pulp percent, and dry matter) changes in olive fruit due to high temperatures in different cultivars (foreign olive cultivars (Konservolia, Sevillana, Manzanilla, and Mission) and Iranian olive cultivars (Shengeh, Roughani, Zard Aliabad, and Dezful)) could provide further insights into the association between the cultivar, oil production, and climatic conditions. Thus, the objective of the present study is to characterize the influence of elevated summer temperatures on the growth characteristics and oil content of eight selected olive cultivars and to identify the best harvesting time for each cultivar among seven different harvesting times. The obtained results will be easily translated into useful practices for the selection of heat-resistant cultivars and of the best harvesting times to improve oil production in changing environmental conditions.

## 2. Results

**Figure 1:**
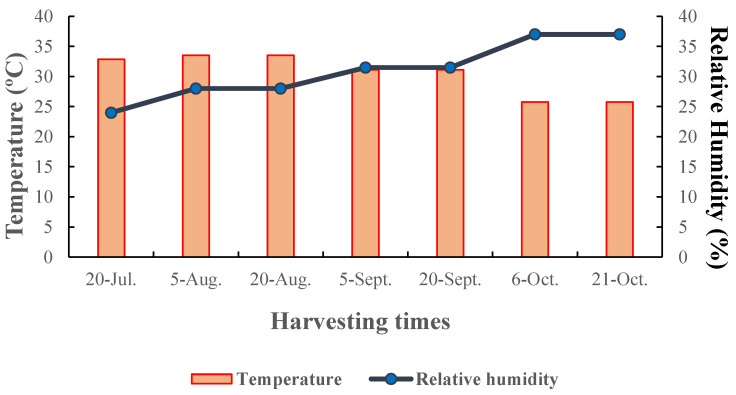
Dynamics of temperature as a first axis accompanied with relative humidity as a second axis during 7 harvesting times.

All of the olive fruit parameters were influenced by the cultivar and harvesting time, with no significant cultivar × harvesting time interaction except for oil content based on the dry and fresh weight and pulp percent. The temperature during the first three harvests was, on average, 33.3 °C, and then it gradually decreased first to a lower steady value of 31.5 °C in September, and a final value of 25.8 °C in the last two harvests in October. The relative humidity, on the other hand, gradually increased from 24% in the first harvest to 37% in the last two harvests (Figure 1). To address the effect of harvesting time and, therefore, temperature, on the olive cultivar yield, the fruit oil content based on dry and fresh weight was monitored in all the different tested olive cultivars from 20 July to 21 October (Table 1). An increasing tendency was found in these parameters, which were positively correlated with fruit growth. However, a significant difference in the rate of oil accumulation among cultivars was also undeniably evident. The results highlight that Roughani presented the highest tested traits irrespective of the other cultivars in approximately all of the harvesting times. Accordingly, the Roughani olive cultivar in the latest harvesting time (21 October) showed the greatest fruit oil accumulation compared to the values exhibited by the other cultivars. On a dry and fresh weight basis, Roughani showed oil values of about 37.67% and 16.96%, respectively, on 21 October. As the mean temperature declined, the oil accumulation in the olive fruits showed an opposite pattern in all of the cultivars. The average daily temperature had a phenomenal effect on oil accumulation, especially in this period of time. In fact, the fruit oil content on a dry and fresh basis progressively increased starting in July, but from 20 September to the last harvesting time, when the temperature decreased from 31.1 °C to 25.8 °C, the oil accumulation showed a maximum slope increase. The fruit oil content appeared to be significantly correlated to dry matter in all single cultivars (0.83 < r < 0.92; *p* < 0.01). The fruit weight and diameter exhibited an increasing tendency during harvesting times, as the relative humidity increased and the temperature decreased, even if there were significant differences between these attributes in the different cultivars. Among the monitored cultivars, Shengeh and Manzanilla showed the highest fruit weights (4.55 and 4.30 g, respectively) and fruit diameters (1.85 and 1.81 cm, respectively) over the different stages (harvesting times), while Konservolia and Mission showed the highest fruit lengths (2.46 and 2.36 cm, respectively). On the contrary, Dezful had the lowest values for the three parameters of fruit weight, length, and diameter (1.90 g, 1.99 cm, and 1.25 cm, respectively) (Table 2).

The fruit volume progressively increased in terms of fruit diameter, weight, and length. Accordingly, the increasing tendency of the fruit volume was recorded during the growth of all olive cultivars. The difference in this trait among the cultivars was significant, and despite Manzanilla showing the greatest fruit volume at the first harvest (3.53 cm^3^) over all other cultivars, Shengeh had the highest fruit volume (5.52 cm^3^) at the end of this study (21 October). The Shengeh cultivar, in fact, significantly grew from 2.98 to 5.52 cm^3^ during the harvest times. The fruit volume of all of the tested cultivars exhibited a steady increase from the beginning of the experiment until September, but in Shengeh, Roughani, Zard Aliabad, Dezful, and Sevillana from 20 August to 5 September, and in Konservolia and Mission from 20 September to 6 October, the fruit volume decreased as the temperature declined. Thus, the mean daily temperature highly affected the fruit volume, too (Table 3). It is worth noting that the fruit pulp on a fresh and dry weight basis also had a remarkable increase during fruit growth, with significant differences among the eight olive cultivars. Although Manzanilla had the greatest values of fresh and dry pulp weight (2.63 and 0.46 g, respectively) on 20 July, Shengeh on 21 October (the final harvesting time) exhibited the highest values of fresh and dry pulp weight (4.48 and 1.11 g, respectively) over all other cultivars (Table 3). Indeed, the pulp growth process continued both at low and high temperatures, although a momentary easing of the fresh and dry pulp growth trend occurred when the temperature started decreasing in September.

The pit weight on a dry and fresh basis similarly showed a significant enhancement from 20 July to 21 October, with significant differences among the cultivars (Table 4). Manzanilla showed the overall highest fresh pit weight and greatest dry pit weight (0.84 and 0.54 g, respectively) over the different cultivars, whereas the Roughani cultivar showed the greatest fresh pit weight (0.91 g) on 21 October over the other cultivars, and Shengeh had the highest dry pit weight at the same harvesting time (0.60 g), even if there was no significant difference between these values and the relative values in the other cultivars at the same harvest time. Generally, the pit lengths of the various cultivars progressively increased during the harvesting times (from 20 July until 21 October), too, and significant differences among the cultivars were observed in the different harvests. Konservolia showed the biggest pit length over all cultivars and harvesting times (Table 4). The fresh pit weight in the olive cultivars revealed a slight reduction in September significantly, but increased again and reached the highest value at the final harvest (Table 4).

The pit diameters, in fact, continually fluctuated in all of the tested olives during the different harvest times (Table 5). However, as for the dry weight, the highest significant values in the pit diameters were recorded in Shengeh and Manzanilla, which, on average, were equal to 0.89 and 0.87, respectively. The latter parameter increased in the eight olive cultivars with a low rate and slow fluctuations during the various stages of development and harvest times (Table 5). Roughani showed the highest value of dry mass (45.0%) in the latest harvest over all the other cultivars and harvest times. The pulp percentage also showed a progressive increase during the harvesting times but with a low slope increase. The highest significant average values of pulp percentage were present in Shengeh and Manzanilla (81.03 and 79.97%, respectively) (Table 5). In Shengeh from July to October, there was an evident but not statistically significant increase in this parameter. The modifications of mean temperature had no distinct effect on the pit diameter of the olive fruits in all of the studied cultivars.

### Cluster Analysis, Heat Map Analysis, and Principal Component Analysis (PCA)

**Figure 2:**
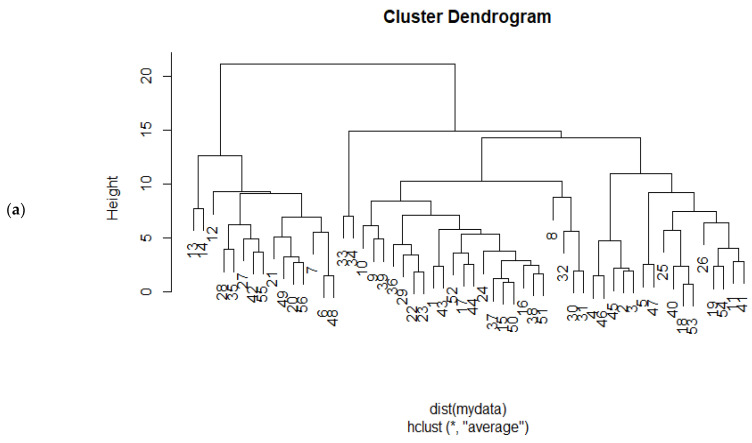

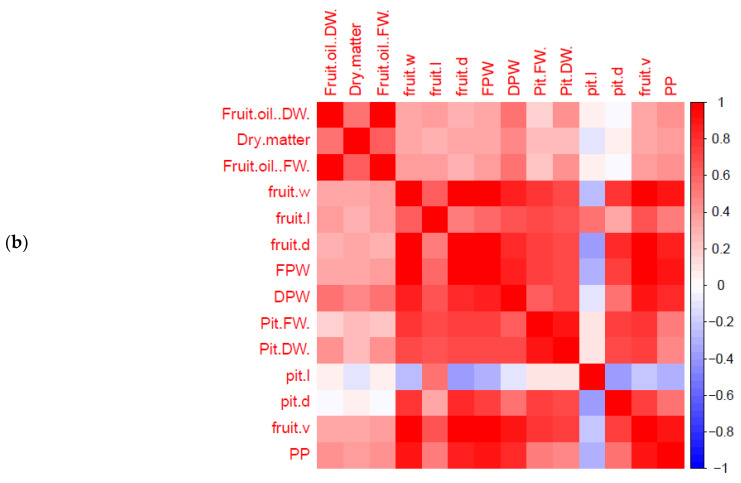
Dendrogram clustering of 7 harvesting times (20 July, 5 and 20 August, 5 and 20 September, 6 and 21 October) in 8 different olive cultivars (Shengeh, Roughani, Konservolia, Zard Aliabad, Dezful, Sevillana, Manzanilla, and Mission) (**a**). Shengeh—20 July, 1; Shengeh—5 August, 2; Shengeh—20 August, 3; Shengeh—5 September, 4; Shengeh—20 September, 5; Shengeh—6 October, 6; Shengeh—21 October, 7; Roughani—20 July, 8; Roughani—5 August, 9; Roughani—20 August, 10; Roughani—5 September, 11; Roughani—20 September, 12; Roughani—6 October, 13; Roughani—21 October, 14; Konservolia—20 July, 15; Konservolia—5 August, 16; Konservolia—20 August, 17; Konservolia—5 September, 18; Konservolia—20 September, 19; Konservolia—6 October, 20; Konservolia—21 October, 21; Zard Aliabad—20 July, 22; Zard Aliabad—5 August, 23; Zard Aliabad—20 August, 24; Zard Aliabad—5 September, 25; Zard Aliabad—20 September, 26; Zard Aliabad—6 October, 27; Zard Aliabad—21 October, 28; Dezful—20 July, 29; Dezful—5 August, 30; Dezful—20 August, 31; Dezful—5 September, 32; Dezful—20 September, 33; Dezful—6 October, 34; Dezful—21 October, 35; Sevillana—20 July, 36; Sevillana—5 August, 37; Sevillana—20 August, 38; Sevillana—5 September, 39; Sevillana—20 September, 40; Sevillana—6 October, 41; Sevillana—21 October, 42; Manzanilla—20 July, 43; Manzanilla—5 August, 44; Manzanilla—20 August, 45; Manzanilla—5 September, 46; Manzanilla—20 September, 47; Manzanilla—6 October, 48; Manzanilla—21 October, 49; Mission—20 July, 50; Mission—5 August, 51; Mission—20 August, 52; Mission—5 September, 53; Mission—20 September, 54; Mission—6 October, 55; Mission—21 October, 56. Heat map of Pearson correlation coefficient summarizing tested variables of 8 different cultivars in 7 harvesting times as treatments (**b**). Positive and negative correlations are exhibited in red and blue colors, respectively, according to the color scale. The abbreviations of olive fruit characteristics in this experiment consist of fruit oil content (%DW), Fruit.oil.DW; fruit oil content (%FW), Fruit.oil.FW; fruit weight, fruit.w; fruit length, fruit.l; fruit diameter, fruit.d; fresh pulp weight, FPW; dry pulp weight, DPW; fresh pit weight, Pit.FW; dry pit weight, Pit.DW; pit length, pit.l; pit diameter, pit.d; fruit volume, fruit.v; pulp percentage, PP.

The cluster dendrogram in Figure 2a shows four groups. The first group includes just Roughani on 6 and 21 October. The second separated group consists of Roughani—20 September; Dezful—21 October; Manzanilla, Zard Aliabad, Konservolia, Shengeh, and Mission—6 and 21 October; and Sevillana—21 October. The third group includes only Dezful—20 September and 6 October. The fourth group, the biggest one, includes Manzanilla—20 July and 5 August; Dezful and Sevillana—5 and 20 August, 5 September, and 20 July; and Roughani, Konservolia, Zard Aliabad, and Mission on 5 and 20 August and 20 July. Ultimately, the fifth group contains Sengeh—5 and 20 September and 5 and 20 August; Manzanilla—5 and 20 September and 20 August; Zard Aliabad, Konservolia, and Mission—5 and 20 September; Sevillana—20 September and 6 October; and Roughani—5 September. The heat map analysis summarizes noticeable positive relationships among the fruit weight, length and diameter, fresh and dry pulp weights, and fresh and dry pit weights. Additionally, between the mentioned attributes and the pit diameter, fruit volume, and pulp percentage. The positive correlations were observed among the fruit oil on a dry and fresh weight basis and dry matter. Conversely, there were remarkable negative relationships between the pit length and fruit diameter, fresh pulp weight, pit diameter, and pulp percentage (Figure 2b).

**Figure 3:**
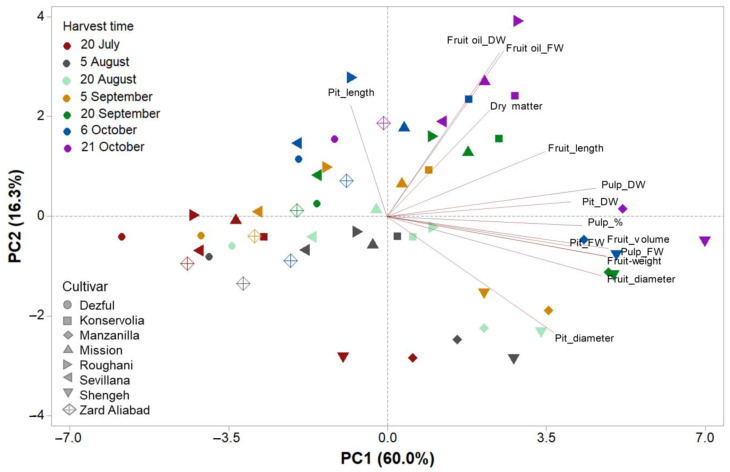
Principal component analysis (PCA) of fruit oil content on a fresh and dry basis and morphological parameters in 8 different elite native and foreign olive cultivars at 7 different harvesting times (related to different temperatures and relative humidity).

A clear separation of cultivars and harvest times was obtained by applying a principal component analysis (PCA). The first two principal components (PCs) were related with eigen values higher than 1 and explained 76.3% of the total variance, with PC1 and PC2 accounting for 60.0% and 16.3%, respectively. The cultivars contributed to the clear separation on PC1, whereas the harvest times contributed to the separation on PC2. The cultivars Shengeh and Manzanilla in the last harvest times were concentrated in the positive side of PC1 and correlated to the fruit volume, fruit weight, fresh and dry pulp weights, pulp percentage, and fresh and dry pit weights, whereas all other cultivars at the first harvesting times were concentrated in the negative side of PC1 and correlated to the pit length. Roughani, at the last harvest time, was clustered in the most positive side of PC2 and was correlated with fruit oil on a dry and fresh weight basis, while Manzanilla and Shengeh at the first harvest were clustered in the negative side of PC2, and correlated to the pit diameter (Figure 3).

## 3. Discussion

The general growth of olive fruit (as a drupe by double sigmoid growth curve) depends on cell division during the early stages of fruit growth, and subsequently, cell expansion dominates after pit hardening. The direct effect of high temperatures during fruit development on the fruit weight is genotype dependent, even if, in our case, the temperature (and relative humidity) affected the fruit growth in all considered cultivars. It was reported that, despite the dry fruit weight at harvest in the Barnea olive cultivar not being affected by a high temperature, the other tested cultivars showed a decrease in the dry fruit weight in response to higher temperatures [5]. So, the Barnea cultivar showed a better tolerance to high temperatures. Also, in our study, two cultivars, Shengeh and Manzanilla, particularly in the last harvest, showed the highest fruit volumes, fruit weights, fresh and dry pulp weights, pulp percentages, and fresh and dry pit weights. Various olive cultivars respond to high temperatures in a genotypic-specific behavior, and the expression level in heat-shock proteins (HSP) is higher in the tolerant cultivar. In Barnea, as a tolerant variety, the ratio between the HSP expression level in high and moderate temperatures was 1308:1, whereas in Souri, a sensitive cultivar, this ratio was 12:1 [5]. In this study, Shengeh, due to having the greatest fruit diameter and volume, fresh and dry pulp weights, dry pit weight, pit diameter, and pulp percentage, proved to be a superior cultivar. The main reasons for the decrease in the pomological characteristics of olives under higher temperatures are attributed to a concomitant increase in the active respiratory rate, oxidative damage, and a loss of photosynthesis efficiency [16]. In this experiment, it can be seen that the fruit growth and development continued until 33.5 °C on 20 August uninterruptedly, in spite of the high temperature in summer. Although it must be noted that starting in July, the relative humidity steadily increased as the temperature decreased. Indeed, these cultivars have high temperature ranges. Thus, the break of fruit development due to the decrease in temperature in the second half of the summer could be ascribed to the fact that the olive plants presumably respond to this change by temporarily reducing their activity and growth, and afterward, they start growing according to the previous growth process again. In the experiment performed in [17], the olive fruit dry weight did not respond to the temperature in the 16–25 °C range; it started decreasing by 0.08 g·°C^−1^ at temperatures beyond 25 °C. Experimental warming declined the rate of fruit growth compared to ambient temperature in both experimental cultivars. This reduction also led to a significant decrease in the final fruit dry weight [18]. In another investigation on olive trees exposed to 37 °C, the reduction in olive shoot and leaf growth was concomitant with low potassium contents and symptoms of dehydration [19].

It seems that the oil content on a fresh weight basis is somewhat unreliable to determine the optimal harvesting time, because it is very sensitive to the tree’s simultaneous water status. Thus, the fluctuations in the fruit water content affect the respective values of the oil content on a fresh weight basis, even if the absolute modifications in the oil content in fruit are negligible [20]. The oil content, on both a dry and fresh weight basis, is controlled by several factors, such as the cultivar, climatic conditions, pulp/pit ratio, etc.; therefore, this trait is also a stress condition indicator [20]. In the present study, as the temperature decreased, and as the relative humidity increased at the end of the summer, the dry matter and oil accumulation on a fresh and dry weight basis suddenly underwent a high slope increase. In particular, Roughani, at the last harvest time, showed the highest fruit oil content on a dry and fresh weight basis. In a previous experiment, an increment in the temperature mitigated the oleic acid content by 0.7% per °C. Moreover, the oil accumulation declined by 1.13% points per °C elevation in the range of 16–32 °C, thus demonstrating that the oil content is sensitive to temperature increases, particularly during the stage in which oil accumulates actively in olive fruits [17]. Sensitive species, which show a decline in oil content, are probably prone to efficiency, due to a high temperature-dependent inhibition of the enzymes involved in chlorophyll biosynthesis [16]. On the other hand, Hemantaranjan et al. [21] showed that, at high temperatures, the reduction in chlorophyll fluorescence in the leaves was inconsistent with the ROS generation due to the excessive absorption of photons, which stimulate the chloroplast and thylakoid membrane disruption [7]. Haworth et al. [22] reported that the decrease in the photosynthetic capacity in olive plants exposed to high temperatures could also be explained by a decrease in Rubisco activity that can reach almost 74% [7]. However, an initial effect of high temperature on several plant species can depend on a decline in the net CO_2_ assimilation rate due to a deficiency of ATP and reducing power (NADPH), which is due to the degradation of photosystem machinery, particularly structural proteins such as D1 protein. In fact, high temperatures [23] can cause an over-reduction in the electron transport chain, inducing photo-inhibitory or photo-oxidative damages to the photosystems [24]. Furthermore, an imbalance between photochemical and biochemical reactions in photosynthesis triggers ROS overproduction and results in oxidative stress, which has a detrimental effect on membrane lipids and photosystems [24]. Regardless of increments in antioxidant activities, electrolyte leakage or MDA content increase upon high-temperature stress [25]. High temperatures (>27 °C) cause a disturbance in the membrane function and integrity, mainly increasing its fluidity while decreasing the electron transport chain’s efficacy, thus determining oxidative burst and lipid peroxidation [26]. Therefore, by increasing the temperature, more than 25–28 °C photosynthesis and other physiological phenomena are decreased, and after that, oil accumulation, as a result of carbohydrate production, has a strong reduction. The latter is even worse, especially when high temperatures are also present during the night, severely increasing respiration and thus decreasing fruit growth, carbohydrate accumulation, and oil formation [7]. 

## 4. Materials and Methods

### 4.1. Experimental Site, Layout, Cultivars, and Harvesting Times

The present study was carried out in Dalahu Olive Research Station of Sarpole Zehab (34°30′ N, 45°51′ E, 581 m above sea level), located in Kermanshah province of Iran, in 2020. Experimental materials of this study were mature trees (20 years old with nearly a 5.5 m height average) of 8 cultivars including 4 foreign olive cultivars (Manzanilla, Sevillana, Konservolia, and Mission) and 4 elite native olive cultivars (Zard Aliabad, Roughani, Dezful, and Shengeh) (Table S1), planted in a 6×6 m distance in a randomized complete block design with three replications (each experimental unit consisted of two trees). Soil and water irrigation samples were analyzed in the soil laboratory of Dalahu Olive Research Station of Sarpole Zehab, and the results with daily meteorological data collected from Sarpole Zehab Synoptic Station in 2020 are presented in Tables S2–S4, respectively. Samples (90 fruits) were randomly taken from each experimental unit from the center and around the tree every two weeks from 20 July to 21 October in 7 different harvesting times (20 July, 5 August, 20 August, 5 September, 20 September, 6 October, 21 October) to find the effect of harvesting time (7 levels) and cultivar (8 levels) on some growth parameters and oil contents of olive trees. The samples were divided into three subsamples of 30 fruits and were washed. 

### 4.2. Growth Parameters

Fruit length and pit length were determined via a Vernier caliper. Fruit and pit diameter were measured via digital Collis. The volume of fruits was measured via transposition by water. Fresh fruit weight, fresh pulp weight (after segregation of its pit), and fresh pit weight (after segregation of fruit pulp) were determined via electrical balance; then, pulp percentage was calculated through the following equation:

$$\mathrm{Pulp}\;\mathrm{percent}\;(\%)=\frac{Fresh\;fruit\;weight-Fresh\;pit\;weight}{Fresh\;fruit\;weight}\;\times \;100$$

Subsequently, the obtained fresh pulps, as well as the fresh pits, were placed in an oven (80 ± 1 °C) for 48 h (or more if needed) [27] to be adequately dried and then weighed using electrical balance to attain dry pulp weight and dry pit weight, respectively.

### 4.3. Fruit Oil Content

The total oil content was determined via Soxhlet extraction using 250 mL of diethyl ether over 8 h [28]. Oil content was calculated based on the fresh and dry weights. The extraction process included fruit crushing and malaxation for 60 min at 35 ± 1 °C with talc and with 100 mL water added between 2 rounds of centrifugation 60 s each at 3000 rpm. After centrifugation, oils were filtered, transferred into amber glass bottles, and stored at 14 °C in the dark.

### 4.4. Statistical Analysis

This study was designed in a completely randomized block in a factorial arrangement with 3 replicates, and each replication included 2 trees (3 × 2 = 6 trees in each treatment) and 2 factors (harvesting times and cultivars; 7 × 8 = 56). The experimental data were analyzed via the two-way analysis of variance (harvesting times and cultivars) using the SAS software (version 9.1, North Carolina). Statistical significance was determined at the p = 0.05 level using Tukey’s honestly significant difference (HSD) test. All data are presented as means, n = 3. Dendrogram clustering was carried out using R.v3.4.3. The loading plot and score plot of all analyzed parameters were determined after principal component analysis (PCA) using Minitab^®^18 statistical software (Minitab LLC, State College, PA, USA) [29]. The associations between analyzed parameters were tested using Pearson correlation analysis.

## 5. Conclusions

This study focuses on the environment–genotype relationships in eight olive cultivars at different harvesting times. The increase in temperature in the summer caused a decrease in the oil content as well as the dry matter, even if the growth restarted in autumn. The results may be useful for predicting possible climatic scenarios with increases in temperatures, even in environments that are currently characterized by a milder climate. Since the Mediterranean has been classified as a climate change hotspot that is projected to undergo an anomalous reduction in winter rainfall (up to 40%) and an increase in average winter and summer temperatures in the next decades, the results showing that the use of various cultivars have a special capacity to cope with high temperatures may be extremely useful in similar circumstances. In particular, Roughani displayed a better tolerance than the other cultivars, but the fruit growth underwent a steady process during the various harvesting times, while Shengeh, which exhibited the better pomological attributes, can be considered as a superior cultivar in relation to yield in a high temperature. Further works under severe climatic conditions would greatly contribute to our insight into the most successful selection of adapted olive cultivars that can tolerate high temperatures.

## Figures and Tables

**Table 1:** Dynamics of fruit oil content on a dry and fresh weight basis as a function of 7 harvesting times in 8 different elite native and foreign olive cultivars.

Source of Variance	Oil Content (% FW)	Oil Content (% DW)
*Cultivar*		
Shengeh	5.74 ± 3.3 bc	15.30 ± 8.4 bcd
Roughani	8.56 ± 5.2 a	20.02 ± 11.4 a
Konservolia	6.06 ± 3.7 b	16.54 ± 9.8 b
Zard Aliabad	5.12 ± 3.1 cde	15.50 ± 8.4 bc
Dezful	4.81 ± 3.1 e	13.87 ± 8.5 de
Sevillana	5.03 ± 3.1 de	13.65 ± 7.9 e
Manzanilla	5.51 ± 3.4 bcd	15.02 ± 8.7 cde
Mission	6.11 ± 3.3 b	16.52 ± 8.5 b
*Harvesting time*		
20-July	1.73 ± 0.4 f	5.09 ± 0.9 f
5-August	2.65 ± 0.5 e	7.49 ± 1.0 e
20-August	3.10 ± 0.6 e	8.71 ± 1.0 e
5-September	5.14 ± 1.4 d	14.10 ± 2.8 d
20-September	7.35 ± 1.6 c	19.62 ± 2.6 c
6-October	9.50 ± 1.7 b	25.25 ± 3.1 b
21-October	11.60 ± 2.1 a	30.37 ± 3.1 a
*Cultivar × Harvesting time*		
Shengeh × 20-July	1.43 ± 0.2 t	4.25 ± 0.3 yz
Shengeh × 5-August	3.06 ± 0.2 pqrst	8.37 ± 0.4 tuvwxyz
Shengeh × 20-August	3.36 ± 0.1 opqrst	9.41 ± 0.4 tuvwxy
Shengeh × 5-September	4.75 ± 0.2 lmnopq	13.07 ± 0.3 qrst
Shengeh × 20-September	7.30 ± 0.4 hijk	19.05 ± 0.6 klmnop
Shengeh × 6-October	9.07 ± 0.4 defgh	24.00 ± 0.9 fghijk
Shengeh × 21-October	11.18 ± 0.4 bcd	28.97 ± 1.6 bcdef
Roughani × 20-July	2.32 ± 0.2 qrst	5.64 ± 0.4 xyz
Roughani × 5-August	3.67 ± 0.3 nopqrst	9.16 ± 0.9 tuvwxyz
Roughani × 20-August	4.22 ± 0.3 mnopqrs	10.17 ± 0.3 stuvwx
Roughani × 5-September	8.35 ± 0.2 efghi	20.40 ± 0.5 jklmnop
Roughani × 20-September	10.81 ± 0.5 cde	24.86 ± 0.9 efghij
Roughani × 6-October	13.61 ± 0.9 b	32.23 ± 1.8 b
Roughani × 21-October	16.96 ± 0.9 a	37.67 ± 1.3 a
Konservolia × 20-July	1.96 ± 0.1 st	5.61 ± 0.4 xyz
Konservolia × 5-August	2.31 ± 0.2 qrst	6.43 ± 0.4 wxyz
Konservolia × 20-August	2.81 ± 0.2 qrst	8.00 ± 0.7 tuvwxyz
Konservolia × 5-September	5.53 ± 0.3 klmnop	15.37 ± 1.1 pqrs
Konservolia × 20-September	7.92 ± 0.3 ghijk	21.33 ± 0.5 jklmno
Konservolia × 6-October	10.25 ± 0.4 cdefg	27.33 ± 1.3 bcdefgh
Konservolia × 21-October	11.66 ± 0.3 bc	31.75 ± 1.2 bc
Zard Aliabad × 20-July	1.88 ± 0.1 st	6.40 ± 0.4 wxyz
Zard Aliabad × 5-August	2.22 ± 0.2 rst	7.30 ± 0.4 vwxyz
Zard Aliabad × 20-August	3.08 ± 0.2 pqrst	9.67 ± 0.7 tuvwx
Zard Aliabad × 5-September	4.11 ± 0.2 mnopqrs	12.83 ± 1.1 qrst
Zard Aliabad × 20-September	5.98 ± 0.1 ijklmn	18.33 ± 0.5 mnop
Zard Aliabad × 6-October	7.90 ± 0.2 ghijk	23.67 ± 1.3 ghijkl
Zard Aliabad × 21-October	10.70 ± 0.3 cdef	30.34 ± 1.2 bcd
Dezful × 20-July	1.37 ± 0.1 t	4.33 ± 0.4 yz
Dezful × 5-August	2.17 ± 0.2 rst	6.57 ± 0.4 wxyz
Dezful × 20-August	2.36 ± 0.3 qrst	7.23 ± 0.7 vwxyz
Dezful × 5-September	3.78 ± 0.4 mnopqrst	11.00 ± 0.8 rstuvw
Dezful × 20-September	5.76 ± 0.4 jklmno	16.34 ± 1.0 opq
Dezful × 6-October	8.23 ± 0.2 fghij	22.84 ± 0.9 hijklm
Dezful × 21-October	10.03 ± 0.3 cdefg	28.80 ± .0.3 bcdefg
Sevillana × 20-July	1.35 ± 0.1 t	4.00 ± 0.0 z
Sevillana × 5-August	2.34 ± 0.2 qrst	6.60 ± 0.5 wxyz
Sevillana × 20-August	2.60 ± 0.1 qrst	7.57 ± 0.4 uvwxyz
Sevillana × 5-September	4.35 ± 0.4 mnopqrs	11.96 ± 0.7 qrstuv
Sevillana × 20-September	6.17 ± 0.2 ijklm	17.00 ± 0.8 nopq
Sevillana × 6-October	8.37 ± 0.2 efghi	21.75 ± 0.5 ijklmn
Sevillana × 21-October	10.05 ± 0.2 cdefg	26.67 ± 0.5 cdefghi
Manzanilla × 20-July	1.41 ± 0.2 t	4.33 ± 0.5 yz
Manzanilla × 5-August	2.61 ± 0.5 qrst	7.37 ± 1.0 vwxyz
Manzanilla × 20-August	2.89 ± 0.5 qrst	8.33 ± 1.0 tuvwxyz
Manzanilla × 5-September	4.55 ± 0.5 mnopqr	12.60 ± 0.6 qrstu
Manzanilla × 20-September	7.05 ± 0.9 hijk	18.70 ± 1.4 lmnop
Manzanilla × 6-October	9.10 ± 0.6 defgh	24.67 ± 1.0 efghij
Manzanilla × 21-October	10.97 ± 0.5 cd	29.17 ± 1.1 bcdef
Mission × 20-July	2.15 ± 0.5 rst	6.17 ± 0.9 wxyz
Mission × 5-August	2.83 ± 0.8 qrst	8.10 ± 1.4 tuvwxyz
Mission × 20-August	3.48 ± 0.7 opqrst	9.33 ± 1.0 tuvwxy
Mission × 5-September	5.72 ± 2.3 klmno	15.60 ± 5.3 pqr
Mission × 20-September	7.82 ± 2.2 ghijk	21.34 ± 4.8 jklmno
Mission × 6-October	9.51 ± 2.5 cdefgh	25.50 ± 4.6 defghij
Mission × 21-October	11.29 ± 1.6 bcd	29.60 ± 2.9 bcde
*Significance*		
*Cultivar*	**	**
*Harvesting time*	**	**
*Cultivar × Harvesting time*	**	**

** indicate significance at *p* ≤ 0.01. Values represent means ± standard deviation of three independent replications (*n* = 3). Different letters within the same column indicate significant differences at *p* < 0.01 among the treatments, according to Tukey’s multiple range test.

**Table 2:** Dynamics of fruit diameter, weight, and length as a function of 7 harvesting times in 8 different elite native and foreign olive cultivars.

Source of Variance	Fruit Weight (g)	Fruit Length (cm)	Fruit Diameter (cm)
*Cultivar*			
Shengeh	4.55 ± 0.8 a	2.21 ± 0.2 bc	1.85 ± 0.2 a
Roughani	2.76 ± 0.6 c	2.07 ± 0.2 d	1.46 ± 0.2 b
Konservolia	3.27 ± 0.5 b	2.46 ± 0.2 a	1.51 ± 0.1 b
Zard Aliabad	2.38 ± 0.4 d	2.00 ± 0.1 d	1.37 ± 0.1 cd
Dezful	1.90 ± 0.4 e	1.99 ± 0.1 d	1.25 ± 0.1 e
Sevillana	2.34 ± 0.4 d	2.08 ± 0.2 cd	1.34 ± 0.1 d
Manzanilla	4.30 ± 0.6 a	2.22 ± 0.1 b	1.81 ± 0.1 a
Mission	3.00 ± 0.5 bc	2.36 ± 0.2 a	1.45 ± 0.1 bc
*Harvesting time*			
20-July	2.19 ± 0.7 e	2.02 ± 0.2 d	1.29 ± 0.2 d
5-August	2.83 ± 0.8 d	2.12 ± 0.2 cd	1.50 ± 0.2 c
20-August	3.16 ± 0.9 bcd	2.20 ± 0.2 bc	1.52 ± 0.3 bc
5-September	2.98 ± 1.0 cd	2.13 ± 0.3 cd	1.50 ± 0.3 bc
20-September	3.38 ± 1.1 ab	2.26 ± 0.3 ab	1.58 ± 0.2 ab
6-October	3.24 ± 1.1 bc	2.17 ± 0.2 bc	1.54 ± 0.3 abc
21-October	3.65 ± 1.0 a	2.32 ± 0.2 a	1.60 ± 0.3 a
*Cultivar × Harvesting time*			
Shengeh × 20-July	3.00 ± 0.3	1.96 ± 0.1	1.56 ± 0.1
Shengeh × 5-August	4.17 ± 0.1	2.09 ± 0.2	1.81 ± 0.1
Shengeh × 20-August	4.82 ± 0.3	2.25 ± 0.1	1.87 ± 0.1
Shengeh × 5-September	4.37 ± 1.0	2.17 ± 0.2	1.88 ± 0.2
Shengeh × 20-September	5.04 ± 0.5	2.36 ± 0.1	1.89 ± 0.1
Shengeh × 6-October	4.85 ± 0.4	2.31 ± 0.2	1.94 ± 0.1
Shengeh × 21-October	5.65 ± 0.5	2.34 ± 0.2	2.00 ± 0.1
Roughani × 20-July	1.72 ± 0.4	1.92 ± 0.4	1.21 ± 0.2
Roughani × 5-August	2.60 ± 0.2	2.04 ± 0.2	1.46 ± 0.1
Roughani × 20-August	3.32 ± 0.3	2.20 ± 0.1	1.62 ± 0.1
Roughani × 5-September	2.59 ± 0.4	1.98 ± 0.2	1.41 ± 0.1
Roughani × 20-September	3.10 ± 0.3	2.10 ± 0.1	1.57 ± 0.1
Roughani × 6-October	2.56 ± 0.3	1.95 ± 0.3	1.45 ± 0.2
Roughani × 21-October	3.46 ± 0.4	2.31 ± 0.2	1.52 ± 0.1
Konservolia × 20-July	2.36 ± 0.5	2.23 ± 0.2	1.30 ± 0.2
Konservolia × 5-August	3.11 ± 0.4	2.42 ± 0.2	1.51 ± 0.1
Konservolia × 20-August	3.31 ± 0.4	2.38 ± 0.2	1.51 ± 0.1
Konservolia × 5-September	3.30 ± 0.3	2.49 ± 0.1	1.51 ± 0.1
Konservolia × 20-September	3.68 ± 0.3	2.64 ± 0.1	1.61 ± 0.1
Konservolia × 6-October	3.53 ± 0.1	2.49 ± 0.1	1.56 ± 0.1
Konservolia × 21-October	3.61 ± 0.1	2.59 ± 0.2	1.57 ± 0.1
Zard Aliabad × 20-July	1.82 ± 0.4	1.94 ± 0.1	1.18 ± 0.1
Zard Aliabad × 5-August	2.18 ± 0.4	1.95 ± 0.2	1.35 ± 0.1
Zard Aliabad × 20-August	2.47 ± 0.3	2.03 ± 0.1	1.36 ± 0.1
Zard Aliabad × 5-September	2.23 ± 0.6	1.93 ± 0.1	1.37 ± 0.1
Zard Aliabad × 20-September	2.45 ± 0.4	1.97 ± 0.2	1.40 ± 0.1
Zard Aliabad × 6-October	2.71 ± 0.3	2.07 ± 0.1	1.44 ± 0.1
Zard Aliabad × 21-October	2.78 ± 0.3	2.16 ± 0.1	1.47 ± 0.1
Dezful × 20-July	1.19 ± 0.1	1.89 ± 0.2	1.03 ± 0.1
Dezful × 5-August	1.72 ± 0.1	1.93 ± 0.1	1.23 ± 0.1
Dezful × 20-August	1.92 ± 0.1	2.04 ± 0.1	1.23 ± 0.1
Dezful × 5-September	1.74 ± 0.1	1.86 ± 0.1	1.23 ± 0.1
Dezful × 20-September	2.16 ± 0.3	2.13 ± 0.1	1.38 ± 0.1
Dezful × 6-October	2.07 ± 0.2	2.07 ± 0.1	1.30 ± 0.0
Dezful × 21-October	2.47 ± 0.3	2.03 ± 0.1	1.34 ± 0.1
Sevillana × 20-July	1.85 ± 0.1	1.97 ± 0.1	1.21 ± 0.1
Sevillana × 5-August	2.31 ± 0.3	2.14 ± 0.2	1.41 ± 0.1
Sevillana × 20-August	2.51 ± 0.2	2.18 ± 0.1	1.37 ± 0.1
Sevillana × 5-September	1.97 ± 0.3	1.99 ± 0.3	1.29 ± 0.1
Sevillana × 20-September	2.39 ± 0.4	2.09 ± 0.2	1.35 ± 0.1
Sevillana × 6-October	2.33 ± 0.5	1.94 ± 0.2	1.31 ± 0.1
Sevillana × 21-October	3.00 ± 0.3	2.28 ± 0.2	1.47 ± 0.1
Manzanilla × 20-July	3.49 ± 1.0	2.09 ± 0.2	1.61 ± 0.2
Manzanilla × 5-August	3.64 ± 0.4	2.09 ± 0.1	1.73 ± 0.1
Manzanilla × 20-August	3.97 ± 0.5	2.21 ± 0.1	1.79 ± 0.1
Manzanilla × 5-September	4.50 ± 0.6	2.22 ± 0.1	1.86 ± 0.1
Manzanilla × 20-September	4.89 ± 0.3	2.33 ± 0.1	1.89 ± 0.1
Manzanilla × 6-October	4.84 ± 0.3	2.23 ± 0.1	1.87 ± 0.1
Manzanilla × 21-October	4.75 ± 0.3	2.38 ± 0.1	1.91 ± 0.2
Mission × 20-July	2.11 ± 0.3	2.18 ± 0.1	1.25 ± 0.1
Mission × 5-August	2.89 ± 0.2	2.34 ± 0.1	1.47 ± 0.1
Mission × 20-August	2.97 ± 0.2	2.35 ± 0.1	1.45 ± 0.1
Mission × 5-September	3.13 ± 0.1	2.37 ± 0.1	1.47 ± 0.1
Mission × 20-September	3.31 ± 0.2	2.49 ± 0.1	1.53 ± 0.1
Mission × 6-October	3.01 ± 0.1	2.29 ± 0.1	1.44 ± 0.1
Mission × 21-October	3.53 ± 0.3	2.51 ± 0.1	1.54 ± 0.1
*Significance*			
*Cultivar*	**	**	**
*Harvesting time*	**	**	**
*Cultivar × Harvesting time*	ns	ns	ns

ns, ** indicate non-significance or significance at *p* ≤ 0.01, respectively. Values represent means ± standard deviation of three independent replications (*n* = 3). Different letters within the same column indicate significant differences at *p* < 0.01 among the treatments, according to Tukey’s multiple range test.

**Table 3:** Dynamics of fruit volume and fresh and dry pulp weights as a function of 7 harvesting times in 8 different elite native and foreign olive cultivars.

Source of Variance	Fresh Pulp Weight (g)	Dry Pulp Weight (g)	Fruit Volume (cm^3^)
*Cultivar*			
Shengeh	3.65 ± 0.7 a	0.75 ± 0.3 a	4.50 ± 0.8 a
Roughani	2.05 ± 0.5 c	0.52 ± 0.2 c	2.74 ± 0.5 cd
Konservolia	2.47 ± 0.4 b	0.60 ± 0.2 bc	3.39 ± 0.5 b
Zard Aliabad	1.72 ± 0.3 d	0.39 ± 0.1 d	2.30 ± 0.3 e
Dezful	1.22 ± 0.4 e	0.35 ± 0.1 d	1.86 ± 0.4 f
Sevillana	1.72 ± 0.3 d	0.57 ± 0.2 c	2.37 ± 0.4 de
Manzanilla	3.46 ± 0.6 a	0.72 ± 0.2 ab	4.29 ± 0.6 a
Mission	2.25 ± 0.4 bc	0.61 ± 0.2 bc	3.09 ± 0.5 bc
*Harvesting time*			
20-July	1.53 ± 0.7 e	0.34 ± 0.1 d	2.21 ± 0.7 e
5-August	2.07 ± 0.7 d	0.51 ± 0.1 c	2.79 ± 0.8 d
20-August	2.37 ± 0.8 bc	0.53 ± 0.2 bc	3.13 ± 0.9 bcd
5-September	2.29 ± 0.9 cd	0.55 ± 0.2 bc	2.98 ± 1.0 cd
20-September	2.60 ± 1.0 ab	0.64 ± 0.2 ab	3.40 ± 1.1 ab
6-October	2.55 ± 1.0 abc	0.64 ± 0.2 ab	3.29 ± 1.1 bc
21-October	2.81 ± 0.9 a	0.74 ± 0.2 a	3.66 ± 1.0 a
*Cultivar × Harvesting time*			
Shengeh × 20-July	2.29 ± 0.2	0.40 ± 0.1	2.98 ± 0.2
Shengeh × 5-August	3.30 ± 0.1	0.57 ± 0.1	4.12 ± 0.2
Shengeh × 20-August	3.85 ± 0.2	0.68 ± 0.1	4.73 ± 0.3
Shengeh × 5-September	3.65 ± 0.6	0.76 ± 0.2	4.27 ± 1.1
Shengeh × 20-September	3.97 ± 0.1	0.85 ± 0.1	4.97 ± 0.5
Shengeh × 6-October	4.01 ± 0.3	0.88 ± 0.2	4.90 ± 0.4
Shengeh × 21-October	4.48 ± 0.1	1.11 ± 0.1	5.52 ± 0.1
Roughani × 20-July	1.12 ± 0.3	0.26 ± 0.1	1.92 ± 0.3
Roughani × 5-August	1.85 ± 0.1	0.62 ± 0.3	2.57 ± 0.2
Roughani × 20-August	2.51 ± 0.2	0.57 ± 0.1	3.24 ± 0.2
Roughani × 5-September	1.99 ± 0.3	0.44 ± 0.1	2.47 ± 0.5
Roughani × 20-September	2.38 ± 0.3	0.59 ± 0.1	3.07 ± 0.4
Roughani × 6-October	1.96 ± 0.3	0.52 ± 0.1	2.50 ± 0.4
Roughani × 21-October	2.55 ± 0.3	0.66 ± 0.1	3.40 ± 0.3
Konservolia × 20-July	1.66 ± 0.4	0.38 ± 0.1	2.41 ± 0.5
Konservolia × 5-August	2.26 ± 0.3	0.54 ± 0.1	3.12 ± 0.3
Konservolia × 20-August	2.48 ± 0.3	0.60 ± 0.1	3.37 ± 0.3
Konservolia × 5-September	2.49 ± 0.3	0.63 ± 0.1	3.47 ± 0.4
Konservolia × 20-September	2.86 ± 0.3	0.65 ± 0.1	3.90 ± 0.3
Konservolia × 6-October	2.82 ± 0.1	0.73 ± 0.1	3.77 ± 0.1
Konservolia × 21-October	2.77 ± 0.1	0.70 ± 0.2	3.72 ± 0.1
Zard Aliabad × 20-July	1.18 ± 0.3	0.29 ± 0.1	1.72 ± 0.3
Zard Aliabad × 5-August	1.51 ± 0.3	0.32 ± 0.1	2.10 ± 0.3
Zard Aliabad × 20-August	1.76 ± 0.3	0.40 ± 0.1	2.40 ± 0.3
Zard Aliabad × 5-September	1.64 ± 0.5	0.37 ± 0.2	2.20 ± 0.5
Zard Aliabad × 20-September	1.85 ± 0.4	0.42 ± 0.2	2.37 ± 0.4
Zard Aliabad × 6-October	2.05 ± 0.2	0.44 ± 0.1	2.63 ± 0.3
Zard Aliabad × 21-October	2.04 ± 0.3	0.49 ± 0.1	2.66 ± 0.3
Dezful × 20-July	0.62 ± 0.1	0.18 ± 0.0	1.16 ± 0.1
Dezful × 5-August	1.06 ± 0.0	0.40 ± 0.3	1.67 ± 0.2
Dezful × 20-August	1.21 ± 0.1	0.28 ± 0.1	1.91 ± 0.1
Dezful × 5-September	1.14 ± 0.1	0.28 ± 0.1	1.69 ± 0.1
Dezful × 20-September	1.41 ± 0.2	0.42 ± 0.1	2.10 ± 0.3
Dezful × 6-October	1.36 ± 0.2	0.38 ± 0.1	2.10 ± 0.1
Dezful × 21-October	1.75 ± 0.3	0.49 ± 0.1	2.41 ± 0.3
Sevillana × 20-July	1.25 ± 0.2	0.37 ± 0.1	1.88 ± 0.1
Sevillana × 5-August	1.74 ± 0.1	0.50 ± 0.1	2.30 ± 0.3
Sevillana × 20-August	1.82 ± 0.2	0.54 ± 0.1	2.40 ± 0.2
Sevillana × 5-September	1.40 ± 0.2	0.52 ± 0.1	1.97 ± 0.3
Sevillana × 20-September	1.80 ± 0.4	0.63 ± 0.1	2.47 ± 0.5
Sevillana × 6-October	1.80 ± 0.4	0.60 ± 0.1	2.47 ± 0.5
Sevillana × 21-October	2.22 ± 0.3	0.82 ± 0.1	3.10 ± 0.3
Manzanilla × 20-July	2.63 ± 0.9	0.46 ± 0.3	3.53 ± 0.9
Manzanilla × 5-August	2.82 ± 0.4	0.62 ± 0.2	3.58 ± 0.4
Manzanilla × 20-August	3.17 ± 0.5	0.66 ± 0.2	3.97 ± 0.5
Manzanilla × 5-September	3.66 ± 0.5	0.79 ± 0.3	4.48 ± 0.5
Manzanilla × 20-September	4.01 ± 0.4	0.81 ± 0.1	4.80 ± 0.3
Manzanilla × 6-October	4.06 ± 0.3	0.88 ± 0.2	4.83 ± 0.4
Manzanilla × 21-October	3.87 ± 0.2	0.82 ± 0.2	4.81 ± 0.3
Mission × 20-July	1.48 ± 0.3	0.37 ± 0.1	2.11 ± 0.3
Mission × 5-August	2.06 ± 0.2	0.52 ± 0.1	2.88 ± 0.1
Mission × 20-August	2.20 ± 0.1	0.54 ± 0.1	3.02 ± 0.2
Mission × 5-September	2.38 ± 0.1	0.60 ± 0.1	3.28 ± 0.1
Mission × 20-September	2.50 ± 0.2	0.73 ± 0.1	3.53 ± 0.3
Mission × 6-October	2.32 ± 0.1	0.67 ± 0.1	3.13 ± 0.2
Mission × 21-October	2.79 ± 0.3	0.84 ± 0.1	3.66 ± 0.3
*Significance*			
*Cultivar*	**	**	**
*Harvesting time*	**	**	**
*Cultivar × Harvesting time*	ns	ns	ns

ns, ** indicate non-significance or significance at *p* ≤ 0.01, respectively. Values represent means ± standard deviation of three independent replications (*n* = 3). Different letters within the same column indicate significant differences at *p* < 0.01 among the treatments, according to Tukey’s multiple range test.

**Table 4:** Dynamics of fresh and dry pit weights and pit length as a function of 7 harvesting times in 8 different elite native and foreign olive cultivars.

Source of Variance	Fresh Pit Weight (g)	Dry Pit Weight (g)	Pit Length (cm)
*Cultivar*			
Shengeh	0.80 ± 0.1 ab	0.51 ± 0.1 ab	1.38 ± 0.1 d
Roughani	0.71 ± 0.2 cde	0.46 ± 0.1 bcd	1.49 ± 0.1 bc
Konservolia	0.79 ± 0.1 abc	0.50 ± 0.1 abc	1.78 ± 0.1 a
Zard Aliabad	0.66 ± 0.1 e	0.45 ± 0.1 cd	1.55 ± 0.1 b
Dezful	0.67 ± 0.1 de	0.46 ± 0.1 bcd	1.58 ± 0.1 b
Sevillana	0.63 ± 0.1 e	0.44 ± 0.1 d	1.56 ± 0.1 b
Manzanilla	0.84 ± 0.1 a	0.54 ± 0.1 a	1.42 ± 0.1 cd
Mission	0.74 ± 0.1 bcd	0.47 ± 0.1 bcd	1.72 ± 0.1 a
*Harvesting time*			
20-July	0.66 ± 0.1 d	0.39 ± 0.1 d	1.55 ± 0.2 ab
5-August	0.76 ± 0.1 ab	0.48 ± 0.1 bc	1.56 ± 0.2 ab
20-August	0.77 ± 0.1 a	0.50 ± 0.1 abc	1.58 ± 0.1 ab
5-September	0.69 ± 0.1 cd	0.45 ± 0.1 c	1.50 ± 0.2 b
20-September	0.75 ± 0.1 abc	0.52 ± 0.1 ab	1.58 ± 0.2 ab
6-October	0.69 ± 0.1 bcd	0.47 ± 0.1 bc	1.53 ± 0.2 ab
21-October	0.81 ± 0.1 a	0.55 ± 0.1 a	1.61 ± 0.2 a
*Cultivar × Harvesting time*			
Shengeh × 20-July	0.70 ± 0.1	0.41 ± 0.1	1.33 ± 0.1
Shengeh × 5-August	0.87 ± 0.1	0.55 ± 0.1	1.37 ± 0.2
Shengeh × 20-August	0.83 ± 0.1	0.52 ± 0.1	1.45 ± 0.2
Shengeh × 5-September	0.73 ± 0.1	0.40 ± 0.1	1.33 ± 0.1
Shengeh × 20-September	0.80 ± 0.2	0.56 ± 0.1	1.40 ± 0.1
Shengeh × 6-October	0.83 ± 0.2	0.55 ± 0.1	1.39 ± 0.1
Shengeh × 21-October	0.88 ± 0.1	0.60 ± 0.1	1.37 ± 0.2
Roughani × 20-July	0.60 ± 0.1	0.35 ± 0.1	1.47 ± 0.2
Roughani × 5-August	0.75 ± 0.1	0.49 ± 0.1	1.51 ± 0.1
Roughani × 20-August	0.81 ± 0.1	0.52 ± 0.1	1.55 ± 0.1
Roughani × 5-September	0.60 ± 0.2	0.39 ± 0.1	1.37 ± 0.2
Roughani × 20-September	0.72 ± 0.1	0.48 ± 0.1	1.45 ± 0.1
Roughani × 6-October	0.60 ± 0.1	0.40 ± 0.1	1.43 ± 0.2
Roughani × 21-October	0.91 ± 0.1	0.59 ± 0.1	1.63 ± 0.1
Konservolia × 20-July	0.66 ± 0.1	0.36 ± 0.1	1.68 ± 0.1
Konservolia × 5-August	0.85 ± 0.1	0.51 ± 0.1	1.77 ± 0.2
Konservolia × 20-August	0.84 ± 0.2	0.52 ± 0.1	1.68 ± 0.2
Konservolia × 5-September	0.81 ± 0.1	0.52 ± 0.1	1.81 ± 0.1
Konservolia × 20-September	0.83 ± 0.1	0.56 ± 0.1	1.89 ± 0.1
Konservolia × 6-October	0.71 ± 0.1	0.48 ± 0.1	1.81 ± 0.1
Konservolia × 21-October	0.85 ± 0.1	0.57 ± 0.1	1.84 ± 0.1
Zard Aliabad × 20-July	0.64 ± 0.1	0.41 ± 0.1	1.59 ± 0.1
Zard Aliabad × 5-August	0.67 ± 0.1	0.44 ± 0.1	1.56 ± 0.1
Zard Aliabad × 20-August	0.71 ± 0.1	0.48 ± 0.1	1.58 ± 0.1
Zard Aliabad × 5-September	0.60 ± 0.1	0.41 ± 0.1	1.47 ± 0.1
Zard Aliabad × 20-September	0.60 ± 0.1	0.43 ± 0.1	1.48 ± 0.2
Zard Aliabad × 6-October	0.67 ± 0.1	0.46 ± 0.1	1.53 ± 0.1
Zard Aliabad × 21-October	0.74 ± 0.1	0.52 ± 0.1	1.63 ± 0.1
Dezful × 20-July	0.57 ± 0.1	0.33 ± 0.1	1.64 ± 0.1
Dezful × 5-August	0.66 ± 0.1	0.44 ± 0.1	1.57 ± 0.1
Dezful × 20-August	0.71 ± 0.1	0.49 ± 0.1	1.65 ± 0.1
Dezful × 5-September	0.60 ± 0.1	0.42 ± 0.1	1.45 ± 0.1
Dezful × 20-September	0.75 ± 0.1	0.55 ± 0.1	1.60 ± 0.1
Dezful × 6-October	0.71 ± 0.1	0.50 ± 0.1	1.59 ± 0.1
Dezful × 21-October	0.72 ± 0.1	0.49 ± 0.1	1.55 ± 0.1
Sevillana × 20-July	0.61 ± 0.1	0.39 ± 0.1	1.55 ± 0.1
Sevillana × 5-August	0.68 ± 0.1	0.46 ± 0.1	1.59 ± 0.1
Sevillana × 20-August	0.68 ± 0.1	0.47 ± 0.1	1.62 ± 0.2
Sevillana × 5-September	0.57 ± 0.1	0.40 ± 0.1	1.51 ± 0.2
Sevillana × 20-September	0.59 ± 0.1	0.44 ± 0.1	1.57 ± 0.1
Sevillana × 6-October	0.54 ± 0.1	0.38 ± 0.1	1.44 ± 0.1
Sevillana × 21-October	0.78 ± 0.1	0.55 ± 0.1	1.65 ± 0.1
Manzanilla × 20-July	0.86 ± 0.2	0.50 ± 0.1	1.48 ± 0.1
Manzanilla × 5-August	0.82 ± 0.1	0.51 ± 0.1	1.41 ± 0.1
Manzanilla × 20-August	0.80 ± 0.1	0.52 ± 0.1	1.42 ± 0.1
Manzanilla × 5-September	0.85 ± 0.2	0.55 ± 0.1	1.37 ± 0.1
Manzanilla × 20-September	0.88 ± 0.1	0.59 ± 0.1	1.44 ± 0.1
Manzanilla × 6-October	0.78 ± 0.1	0.52 ± 0.1	1.37 ± 0.2
Manzanilla × 21-October	0.88 ± 0.2	0.59 ± 0.1	1.45 ± 0.1
Mission × 20-July	0.62 ± 0.1	0.37 ± 0.1	1.65 ± 0.1
Mission × 5-August	0.83 ± 0.1	0.49 ± 0.1	1.73 ± 0.2
Mission × 20-August	0.77 ± 0.1	0.47 ± 0.1	1.72 ± 0.1
Mission × 5-September	0.75 ± 0.1	0.48 ± 0.1	1.70 ± 0.2
Mission × 20-September	0.82 ± 0.1	0.55 ± 0.1	1.81 ± 0.1
Mission × 6-October	0.69 ± 0.2	0.46 ± 0.1	1.66 ± 0.1
Mission × 21-October	0.74 ± 0.0	0.50 ± 0.1	1.77 ± 0.1
*Significance*			
*Cultivar*	**	**	**
*Harvesting time*	**	**	**
*Cultivar × Harvesting time*	ns	ns	ns

ns, ** indicate non-significance or significance at *p* ≤ 0.01, respectively. Values represent means ± standard deviation of three independent replications (*n* = 3). Different letters within the same column indicate significant differences at *p* < 0.01 among the treatments, according to Tukey’s multiple range test.

**Table 5:** Dynamics of pit diameter, dry matter, and pulp percentage as a function of 7 harvesting time in 8 different elite native and foreign olive cultivars.

Source of Variance	Dry Matter (%)	Pit Diameter (cm)	Pulp Percent (%)
*Cultivar*			
Shengeh	36.72 ± 1.6 b	0.89 ± 0.1 a	81.03 ± 2.7 a
Roughani	42.01 ± 1.6 a	0.78 ± 0.1 b	73.63 ± 4.2 bcd
Konservolia	36.22 ± 0.9 b	0.79 ± 0.1 b	75.28 ± 3.0 b
Zard Aliabad	32.15 ± 1.8 d	0.77 ± 0.1 b	71.97 ± 2.7 d
Dezful	33.95 ± 1.5 c	0.78 ± 0.1 b	66.00 ± 3.2 e
Sevillana	36.06 ± 1.6 b	0.77 ± 0.1 b	72.48 ± 3.0 cd
Manzanilla	35.77 ± 1.8 b	0.87 ± 0.1 a	79.97 ± 2.9 a
Mission	36.21 ± 1.2 b	0.79 ± 0.1 b	74.73 ± 3.0 bc
*Harvesting time*			
20-July	33.95 ± 3.2 d	0.78 ± 0.1 c	70.14 ± 3.7 d
5-August	35.13 ± 2.6 cd	0.83 ± 0.1 a	71.56 ± 5.1 d
20-August	35.36 ± 2.8 cd	0.81 ± 0.1 abc	73.89 ± 5.1 c
5-September	36.00 ± 2.4 bc	0.78 ± 0.1 c	74.84 ± 4.8 bc
20-September	37.13 ± 2.9 ab	0.82 ± 0.1 a	76.38 ± 5.3 ab
6-October	37.40 ± 2.4 ab	0.79 ± 0.1 bc	77.28 ± 5.2 a
21-October	37.98 ± 3.0 a	0.81 ± 0.1 ab	76.61 ± 4.4 ab
*Cultivar × Harvesting time*			
Shengeh × 20-July	33.63 ± 0.7	0.87 ± 0.1	76.57 ± 1.1 abcdefghijklmn
Shengeh × 5-August	36.52 ± 0.5	0.93 ± 0.1	79.20 ± 0.7 abcdefghijk
Shengeh × 20-August	35.80 ± 0.9	0.88 ± 0.0	80.11 ± 3.2 abcdefgh
Shengeh × 5-September	36.39 ± 1.2	0.80 ± 0.1	79.90 ± 2.3 abcdefghi
Shengeh × 20-September	38.28 ± 0.7	0.91 ± 0.1	84.13 ± 1.1 ab
Shengeh × 6-October	37.76 ± 0.4	0.90 ± 0.1	82.84 ± 1.7 abc
Shengeh × 21-October	38.65 ± 0.9	0.93 ± 0.1	84.43 ± 0.6 a
Roughani × 20-July	41.02 ± 0.5	0.75 ± 0.1	64.69 ± 2.6 rst
Roughani × 5-August	40.05 ± 0.6	0.81 ± 0.1	71.13 ± 1.8 ijklmnopqrs
Roughani × 20-August	41.40 ± 1.4	0.81 ± 0.1	75.57 ± 1.8 abcdefghijklmno
Roughani × 5-September	40.92 ± 0.9	0.76 ± 0.0	77.09 ± 2.0 abcdefghijklm
Roughani × 20-September	43.49 ± 0.5	0.80 ± 0.1	76.82 ± 1.5 abcdefghijklm
Roughani × 6-October	42.22 ± 0.9	0.73 ± 0.1	76.54 ± 3.0 abcdefghijklmn
Roughani × 21-October	45.00 ± 0.9	0.79 ± 0.1	73.58 ± 1.4 efghijklmnopq
Konservolia × 20-July	35.03 ± 0.6	0.79 ± 0.1	70.10 ± 3.5 mnopqrst
Konservolia × 5-August	35.91 ± 0.9	0.81 ± 0.1	72.62 ± 2.0 fghijklmnopqr
Konservolia × 20-August	35.16 ± 0.5	0.78 ± 0.1	74.90 ± 1.7 defghijklmno
Konservolia × 5-September	36.05 ± 0.6	0.77 ± 0.1	75.45 ± 1.1 bcdefghijklmno
Konservolia × 20-September	37.13 ± 0.5	0.81 ± 0.1	77.39 ± 2.5 abcdefghijklm
Konservolia × 6-October	37.54 ± 1.1	0.77 ± 0.1	79.86 ± 0.5 abcdefghi
Konservolia × 21-October	36.75 ± 0.6	0.83 ± 0.1	76.59 ± 1.6 abcdefghijklmn
Zard Aliabad × 20-July	29.43 ± 0.9	0.75 ± 0.1	67.79 ± 0.2 nopqrst
Zard Aliabad × 5-August	30.37 ± 1.0	0.79 ± 0.1	68.90 ± 3.8 mnopqrst
Zard Aliabad × 20-August	31.92 ± 1.1	0.78 ± 0.1	71.03 ± 3.6 ijklmnopqrs
Zard Aliabad × 5-September	32.02 ± 1.2	0.75 ± 0.1	72.43 ± 4.2 ghijklmnopqr
Zard Aliabad × 20-September	32.67 ± 1.3	0.77 ± 0.1	75.25 ± 2.7 cdefghijklmno
Zard Aliabad × 6-October	33.38 ± 0.6	0.79 ± 0.1	75.31 ± 0.6 bcdefghijklmno
Zard Aliabad × 21-October	35.25 ± 0.4	0.78 ± 0.1	73.12 ± 2.6 efghijklmnopqr
Dezful × 20-July	31.60 ± 1.0	0.73 ± 0.1	70.00 ± 0.0 mnopqrst
Dezful × 5-August	33.07 ± 1.0	0.80 ± 0.0	61.48 ± 1.7 t
Dezful × 20-August	32.52 ± 1.0	0.79 ± 0.1	63.12 ± 2.2 st
Dezful × 5-September	34.28 ± 0.7	0.77 ± 0.1	65.43 ± 1.6 qrst
Dezful × 20-September	35.26 ± 0.9	0.83 ± 0.1	65.33 ± 1.3 qrst
Dezful × 6-October	36.06 ± 1.2	0.81 ± 0.1	65.86 ± 0.2 pqrst
Dezful × 21-October	34.83 ± 0.6	0.77 ± 0.1	70.78 ± 2.8 jklmnopqrs
Sevillana × 20-July	33.78 ± 0.9	0.75 ± 0.1	67.12 ± 3.0 opqrst
Sevillana × 5-August	35.41 ± 0.6	0.81 ± 0.1	70.54 ± 0.4 klmnopqrs
Sevillana × 20-August	34.45 ± 0.8	0.78 ± 0.0	72.73 ± 0.1 fghijklmnopqr
Sevillana × 5-September	36.29 ± 0.7	0.78 ± 0.1	71.33 ± 0.7 hijklmnopqrs
Sevillana × 20-September	36.33 ± 0.6	0.76 ± 0.1	74.96 ± 3.2 cdefghijklmno
Sevillana × 6-October	38.51 ± 0.7	0.72 ± 0.1	76.76 ± 2.9 abcdefghijklm
Sevillana × 21-October	37.67 ± 0.3	0.79 ± 0.1	73.92 ± 1.7 efghijklmnopq
Manzanilla × 20-July	32.49 ± 0.6	0.89 ± 0.1	74.70 ± 2.8 defghijklmnop
Manzanilla × 5-August	35.22 ± 1.4	0.90 ± 0.1	77.25 ± 2.1 abcdefghijklm
Manzanilla × 20-August	34.56 ± 1.5	0.87 ± 0.1	79.65 ± 1.8 abcdefghij
Manzanilla × 5-September	36.01 ± 2.2	0.86 ± 0.0	81.13 ± 3.5 abcdefg
Manzanilla × 20-September	37.61 ± 1.8	0.89 ± 0.1	81.78 ± 1.8 abcde
Manzanilla × 6-October	36.85 ± 1.4	0.84 ± 0.1	83.80 ± 1.9 abc
Manzanilla × 21-October	37.65 ± 1.8	0.88 ± 0.1	81.46 ± 2.0 abcdef
Mission × 20-July	34.63 ± 4.7	0.75 ± 0.1	70.16 ± 2.2 lmnopqrst
Mission × 5-August	34.48 ± 3.7	0.83 ± 0.1	71.34 ± 1.6 hijklmnopqrs
Mission × 20-August	37.10 ± 4.0	0.79 ± 0.1	74.01 ± 1.6 defghijklmnopq
Mission × 5-September	36.08 ± 2.3	0.78 ± 0.1	75.94 ± 2.6 abcdefghijklmno
Mission × 20-September	36.28 ± 2.0	0.83 ± 0.1	75.36 ± 2.0 bcdefghijklmno
Mission × 6-October	36.88 ± 3.2	0.77 ± 0.1	77.25 ± 3.3 abcdefghijklm
Mission × 21-October	38.03 ± 2.5	0.77 ± 0.1	79.02 ± 1.4 abcdefghijkl
*Significance*			
*Cultivar*	**	**	**
*Harvesting time*	**	**	**
*Cultivar × Harvesting time*	ns	ns	**

ns, ** indicate non-significance or significance at *p* ≤ 0.01, respectively. Values represent means ± standard deviation of three independent replications (*n* = 3). Different letters within the same column indicate significant differences at *p* < 0.01 among the treatments, according to Tukey’s multiple range test.

## Data Availability

The datasets generated and analyzed during the current study are available from the corresponding author upon reasonable request.

## Supplementary Materials

The following supporting information can be downloaded at: https://www.mdpi.com/article/10.3390/plants12142737/s1, Table S1: Main characteristics of native and foreign olive cultivars undergoing water shortage in the Dallaho Olive Research Station in Sarpol-Zahab city, Kermanshah Province; Table S2: Physical and chemical characteristics of the experimental soil (Kermanshah Laboratory of Soil Science, Agricultural Research and Education Center); Table S3: Characteristics of water Irrigation (Kermanshah Laboratory of Soil Science, Agricultural Research and Education Center); Table S4: Average monthly temperature, relative humidity, evaporation and rainfall of Sarpol-e zahab (2020).
