# Supplementary material for: Effects of Harvesting Time on Fruit Development Process and Oil Content of Selected Iranian and Foreign Olive Cultivars under Subtropical Conditions
Source: Plants (Basel). 2023 Jul 23;12(14):2737. doi: 10.3390/plants12142737 (PMC10385431; doi:10.3390/plants12142737)
Supplement: Supplementary file 1 [file plants-12-02737-s001.zip › plants-2479934-supplementary.pdf]

Table S1. Main characteristics of native and foreign olive cultivars undergoing water shortage in the Dallaho Olive Research Station in Sarpol-Zahab city, Kermanshah Province

Cultivar	Original country	Commercial use	Fruit ripening time	Growth characteristic
Manzanilla	Spain	conserved	Late November	-Wide-spreading growth habit
				-high canopy density
Sevillana	Spain	conserved	Early December	-stand growth habit
				-moderate canopy density
Mission	America	Dual purpose	Early December	-stand growth habit
				-high canopy density
Konservolia	Greece	conserved	Mid October	-stand growth habit
				-moderate canopy density
Zard Aliabad	Iran	Dual purpose	Mid October	Wide-spreading growth habit
				-high canopy density
Roughani	Iran	Oil production	Early October	-stand growth habit
				-moderate canopy density
Dezful	Iran	conserved	Mid October	-stand growth habit
				-moderate canopy density
Shengeh	Iran	conserved	Mid October	-Wide-spreading growth habit
				-high canopy density

Table S2. Physical and chemical characteristics of the experimental soil (Kermanshah Laboratory of Soil Science, Agricultural Research and Education Center)

Sample	Soil depth (cm)	pH	P^+ (ppm)	K^+ (ppm)	Saturation (%)	$CaCO_3$ (%)	Organic carbon (%)	nitrogen (%)	Sand (%)	Silt (%)
1	0-30	7.30	11.80	320	40	33	1.95	0.17	24	44
2	30-60	7.50	6	100	49	35	1.20	0.05	27	37

Table S3. Characteristics of water Irrigation (Kermanshah Laboratory of Soil Science, Agricultural Research and Education Center)

EC (mmhos.cm^{-1})	Total soluble salts (mg.L^{-1})	Carbonate (meq.L^{-1})	Bicarbonate (meq.L^{-1})	Cholorine (meq.L^{-1})	Sulfate (meq.L^{-1})	Calcium (meq.L^{-1})	Sodium (meq.L^{-1})	pH
550	352	0	4.60	0.30	1.90	6.60	0.20	7.28

Table S4. Average monthly temperature, relative humidity, evaporation and rainfall of Sarpol-e zahab (2020)

Month	Maximum temperature (°C)	Minimum temperature (°C)	Minimum relative humidity (%)	Maximum relative humidity (%)	Rainfall (mm)
May	31.5	14.2	24	65	2.7
Jun	38.8	18.4	12	42	0
July	42.2	23.5	12	36	0
Aug	42.8	24.2	13	42	0
Sep	41	21.2	13	38	0.5
Oct	35.1	16.4	14	45	0
